# Evaluating the Accuracy of Responses by Large Language Models for Information on Disease Epidemiology

**DOI:** 10.1002/pds.70111

**Published:** 2025-02-03

**Authors:** Kexin Zhu, Jiajie Zhang, Anton Klishin, Mario Esser, William A. Blumentals, Juhaeri Juhaeri, Corinne Jouquelet‐Royer, Sarah‐Jo Sinnott

**Affiliations:** ^1^ Epidemiology and Benefit Risk, Sanofi Bridgewater New Jersey USA; ^2^ Babraham Research Campus Sanofi Cambridge UK; ^3^ Global Pharmacovigilance Sanofi Warsaw Poland; ^4^ Global Pharmacovigilance Sanofi Frankfurt Germany; ^5^ Epidemiology and Benefit Risk Sanofi Cambridge Massachusetts USA; ^6^ Global Pharmacovigilance Sanofi Paris France

**Keywords:** artificial intelligence, ChatGPT, Google Bard, large language model, pharmacoepidemiology

## Abstract

**Purpose:**

Accurate background epidemiology of diseases are required in pharmacoepidemiologic research. We evaluated the performance of large language models (LLMs), including ChatGPT‐3.5, ChatGPT‐4, and Google Bard, when prompted with questions on disease frequency.

**Methods:**

A total of 21 questions on the prevalence and incidence of common and rare diseases were developed and submitted to each LLM twice on different dates. Benchmark data were obtained from literature searches targeting “gold‐standard” references (e.g., government statistics, peer‐reviewed articles). Accuracy was evaluated by comparing LLMs' responses to the benchmark data. Consistency was determined by comparing the responses to the same query submitted on different dates. The relevance and authenticity of references were evaluated.

**Results:**

Three LLMs generated 126 responses. In ChatGPT‐4, 76.2% of responses were accurate, which was higher compared to 50.0% in Bard and 45.2% in ChatGPT‐3.5. ChatGPT‐4 exhibited higher consistency (71.4%) than Bard (57.9%) or ChatGPT‐3.5 (46.7%). ChatGPT‐4 provided 52 references with 27 (51.9%) providing relevant information, and all were authentic. Only 9.2% (10/109) of references from Bard were relevant. Of 65/109 unique references, 67.7% were authentic, 7.7% provided insufficient information for access, 10.8% provided inaccurate citation, and 13.8% were non‐existent/fabricated. ChatGPT‐3.5 did not provide any references.

**Conclusions:**

ChatGPT‐4 outperformed in retrieving information on disease epidemiology compared to Bard and ChatGPT‐3.5. However, all three LLMs presented inaccurate responses, including irrelevant, incomplete, or fabricated references. Such limitations preclude the utility of the current forms of LLMs in obtaining accurate disease epidemiology by researchers in the pharmaceutical industry, in academia, or in the regulatory setting.

**Summary**

- ChatGPT‐4 presented higher accuracy and consistency than Google Bard and ChatGPT‐3.5 in responding to questions on disease epidemiology when compared with benchmark data identified from a traditional literature search.
- All three LLMs presented inaccuracies in their responses, including irrelevant, incomplete, or fabricated references.
- The accuracy and reliability of LLMs' responses are not of acceptable standards for researchers in establishing background epidemiology of diseases for use in drug safety research.

## 1 Introduction

Large language models (LLMs) represent a significant advancement in the field of generative artificial intelligence (AI) and natural language processing. LLM‐based chatbots, such as ChatGPT (Open AI, San Francisco, CA) and Bard (Google, Mountain View, CA), have gained popularity due to their ability to generate high‐quality human‐like responses related to the context of the input across various domains [1, 2, 3]. There has been a tremendous increase in the use of LLMs in the scientific community, with many researchers using the chatbots in a variety of tasks, such as literature review, manuscript drafting and polishing, and statistical analyses [4, 5, 6, 7]. However, there are concerns about incorrect or fake information, that is seemingly scientifically plausible, in LLMs' responses [8, 9]. Recently, ChatGPT has been found to create a fake data set to support an unverified scientific hypothesis [9]. In addition, issues have been identified regarding citation inaccuracies and insufficiency, or even referencing non‐existent sources, providing further indication for fabricated data [10, 11]. These drawbacks pose significant challenges in the application and the trust of LLMs in many scientific disciplines, including the field of pharmacoepidemiology, which relies on accurate data to support various aspects of drug development inclusive of drug safety research.

In post‐marketing drug safety research, a typical question to address is whether a medical event is occurring at a higher rate in a drug exposed population than in a similar non‐drug exposed population. A higher rate in the drug‐exposed population may indicate a potential safety signal for the drug in question. Accurate background epidemiology of diseases from independent sources, including published epidemiological studies, national statistics, or *de* 20 *novo* database analyses, play a critical role in these scenarios by providing contextual data for how often a particular health event should be occurring in a particular population. These “expected” rates can then be compared to the “observed” rates in the drug exposed population [12]. The exercise hinges on the utility of high quality, up‐to‐date and transparently procured data [13, 14]. The need for rapid data on background rates of diseases, for example, pericarditis amongst many others, was well demonstrated by the COVID‐19 pandemic, where the safety of rapidly developed vaccines had to be assessed in a highly visible, highly sensitive real‐world setting [15, 16]. On one hand, given LLM's ability in gathering and summarizing information from extensive online sources, it may be a useful tool to expedite the retrieval of existing data on disease epidemiology. On the other hand, the use of inaccurate epidemiology data may jeopardize the quality of drug safety evaluations, consequently undermining trust in rigorous and highly regulated safety procedures and ultimately impacting safety of patients [17]. It is not yet known what the potential of LLMs is for providing reliable and accurate pharmacoepidemiologic data that could be of utility in drug safety research.

In this study, we aimed to evaluate the accuracy and consistency of information provided by LLMs on questions related to disease occurrence (prevalence and incidence of common and rare diseases). In addition, we aimed to evaluate the relevance and authenticity of references provided in LLMs' responses.

## 2 Methods

### 2.1 Study Design and Setting

This was a cross‐sectional study. We developed a set of 21 questions on typical epidemiological measures of disease frequency: incidence and prevalence. These questions were submitted into three LLMs: ChatGPT‐3.5 [1], ChatGPT‐4 [1], and Google Bard [2]. The accuracy of responses was compared with benchmark data identified through traditional literature searches. Each question was submitted to each LLM twice on different dates (two queries for each question in three tools) to evaluate the consistency of responses to the same questions. The quality of references provided in the responses were also evaluated in terms of authenticity and relevance. The detailed methods are described below.

### 2.2 Questions on the Measures of Disease Frequency

Two researchers (K.Z. and S.J.S.) designed a total of 21 open‐ended questions on the prevalence and incidence for two common diseases (hypertension and diabetes) and two rare diseases (amyotrophic lateral sclerosis and laryngeal cancer) (Table S1). In the United States, a rare disease is defined by the 1983 Orphan Drug Act as a disease or condition that affects less than 200 000 people [18]. We selected rare diseases for which we could obtain benchmark evidence from reliable sources. The questions covered measures of prevalence and incidence of diseases in different time periods and sub‐populations, for example age and sex sub‐groups. We limited the geographic region to the United States to reduce the burden of manual search for existing benchmark evidence. We also limited the time periods prior to 2021 because ChatGPT was trained based on data limited up to September 2021 at the time we conducted this analysis [1].

### 2.3 Benchmark Data From Traditional Literature Search

A researcher (K.Z.) performed literature searches to find benchmark data for disease occurrence. The literature search targeted known “gold standard” reference materials (e.g., government statistics and epidemiological data, disease treatment guidelines from reputable professional societies including American Heart Association) along with PubMed, and Google Scholar searches. All answers on disease occurrence were reviewed and verified by the second researcher (S.J.S.).

### 2.4 Data Collection Using ChatGPT and Google Bard

Three LLMs, ChatGPT‐3.5, ChatGPT‐4, and Google Bard were used. ChatGPT 3.5 and Google Bard can be accessed by the public for free, while ChatGPT‐4 is available only through a paid subscription. A single researcher (K.Z.) inputted all the questions into the LLM‐based chatbots. A new chat session was created for each question to prevent previous output for a particular question from influencing the model's response. To assess the consistency of the responses, each question was re‐submitted to each LLM on a different date after the initial query by the same researcher (K.Z.). Data were collected between October 31 and November 17, 2023.

### 2.5 Performance Evaluation

The responses from LLMs were compared to answers from “benchmark” data retrieved from manual searches. To determine accuracy, we categorized LLMs' responses to each question as correct, somewhat correct (the response was close to but not exactly the same as the correct answer, with a ±10%‐difference allowed), incorrect, or unanswered (LLMs did not provide responses or did not know the answers). The accuracy for each LLM was then calculated as a proportion, that is, the numerator was the number of correct/somewhat correct responses and the denominator was the total number of responses. The consistency of responses was evaluated to examine if LLMs could provide consistent responses to the same question at two different time points (±10%‐difference allowed). The consistency of responses for each LLM was calculated as a proportion, that is, the numerator was the number of consistent responses and the denominator was the number of questions after removing unanswered questions in both queries in each LLM. In addition, we evaluated the quality of references in terms of relevance and authenticity. Relevance was evaluated if the content in the reference source was specific to the question submitted to LLMs and provided enough information for generating correct responses. We evaluated authenticity in the unique references after removing duplicated references, and categorized references as: (1) authentic (accurate “real” references identifiable via internet search), (2) incomplete (insufficient information to verify its existence), (3) inaccurate (identifiable through internet search but one or more elements in citations were incorrect, e.g., journal names, volume or page numbers), and (4) fabricated (made‐up references).

### 2.6 Statistical Analysis

Categorical variables were presented by counts and proportions. For each LLM, the accuracy of responses was calculated as the proportion of correct or somewhat correct responses compared to results from benchmark data. The consistency of responses for each LLM was determined by the proportion of consistent responses for the same query at two time points. The differences in the accuracy and consistency of responses from three LLMs were compared using two‐sided chi‐square or Fisher's exact tests. All statistical analyses were performed using R version 4.2.3.

## 3 Results

**FIGURE 1:**
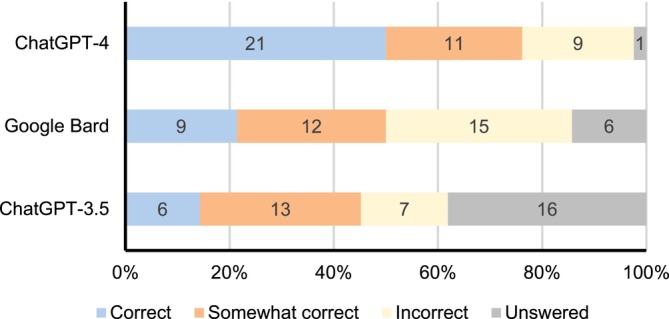
Evaluation of responses from ChatGPT‐4, Google Bard, and ChatGPT‐3.5 compared to benchmark data obtained from literature searches. Data label in chart refers to the number of correct, somewhat correct, incorrect, or unanswered responses from each large language model compared to benchmark data obtained from literature searches.

**FIGURE 2:**
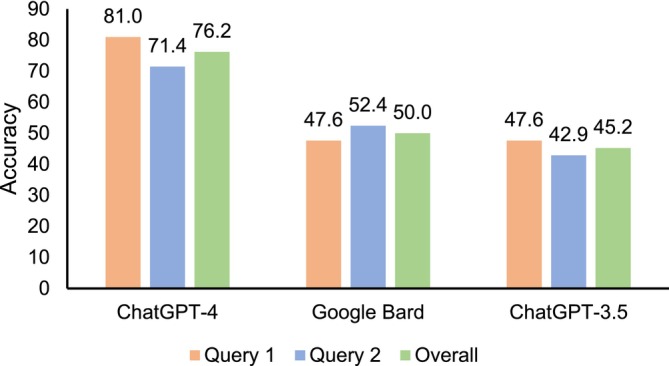
Accuracy of responses from ChatGPT‐4, Google Bard, and ChatGPT‐3.5 overall and in two queries on two different dates. Accuracy is calculated as the sum of the number of responses rated as “Correct” or “Somewhat correct” divided by the total number of responses in each query (*N* = 21) or overall (*N* = 42 responses in two queries).

We developed 21 questions, among which 11 covered disease frequency for common diseases and 10 for rare diseases. All responses from LLMs are documented in Table S2 and results from the traditional literature searches are presented in Table S3. Each LLM generated 42 responses after each question was submitted at two distinct time points, resulting in 126 responses evaluated. Figure 1 and Table S4 present the overall accuracy of responses from LLMs compared to results obtained from traditional literature searches. In ChatGPT‐4, 76.2% (32/42) of the responses were correct or somewhat correct, which was higher compared to 50.0% (21/42) in Google Bard (*p* = 0.030) or 45.2% (19/42) in ChatGPT‐3.5 (*p* = 0.001). Only 2.4% of responses provided by ChatGPT‐4 did not answer the questions, while 14.3% of responses in Google Bard and 38.1% in ChatGPT‐3.5 failed to provide information related to the questions. We further compared accuracy of responses by disease types for each LLM (Table S5). The accuracy for common diseases was higher than that for rare diseases in three LLMs (ChatGPT‐3.5: 72.7% vs. 15.0%, *p* < 0.001; ChatGPT‐4: 81.8% vs. 70.0%, *p* = 0.477; Google Bard: 63.6% vs. 35.0%, *p* = 0.064). We also found that the accuracy of responses did not differ significantly in the same queries on two different dates for each LLM (Figure 2 and Table S6). The consistency of responses in queries at two different times for ChatGPT‐4, Google Bard, and ChatGPT‐3.5 was 71.4%, 57.9%, and 46.7%, respectively (Table S7), after removing unanswered responses in both queries.

In ChatGPT‐4, a total of 52 references were generated for 21 questions applied twice, of which 27 (51.9%) provided relevant information to correctly answer the questions (Table S8). After removing duplicates from the 52 references, we analyzed the authenticity of 20 unique references provided by ChatGPT‐4 (Table 1). All the references could be accessed through internet searches (real references): 8 (40.0%) were found in the PubMed database and 12 (60.0%) consisted of hyperlinks to web pages. Google Bard generated 109 references in total, with only 10 (9.2%) considered to provide information to correctly answer the questions (Table S9). Among 65 unique references after removing duplicated references, the majority (*n* = 44, 67.7%) were real references; however, 5 (7.7%) were incomplete, 7 (10.8%) were inaccurate, and 9 were fabricated references (13.8%) (Table 1). Examples of incomplete, inaccurate, and fabricated references found in Google Bard's responses are shown in Table 2. ChatGPT‐3.5 did not generate any references.

**TABLE 1:** Analysis of references provided in responses of ChatGPT‐4 and Google Bard.

Category	ChatGPT‐4	Google Bard
Total number of references	52	109
Relevant^a^, *n* (%)	27 (51.9)	10 (9.2)
Irrelevant^a^, *n* (%)	25 (48.1)	99 (90.8)
Total number of unique references	20	65
Authentic/accurate (real reference)^b^, *n* (%)	20 (100)	44 (67.7)
PubMed	8 (40.0)^c^	4 (9.1)^c^
Hyperlinks to web pages	12 (60.0)^c^	40 (90.9)^c^
Incomplete^d^, *n* (%)	0 (0)	5 (7.7)
Inaccurate^e^, *n* (%)	0 (0)	7 (10.8)
Fabricated, *n* (%)	0 (0)	9 (13.8)

^a^ Relevance was evaluated if the content in the reference was specific to the question submitted to large language models and provided enough information for generating correct responses.

^b^ Real reference identifiable via internet search.

^c^ The denominator was the total number of authentic/accurate references.

^d^ References without sufficient information to verify existence.

^e^ References identifiable via internet search but with incorrect author names, journal names, volume or page numbers, DOI, or hyperlinks.

**TABLE 2:** Examples of problematic references in Google Bard's responses.

Category	Reference	Comments
Irrelevant^a^	Mayo Clinic. High Blood Pressure (Hypertension). Accessed November 17, 2023. https://www.mayoclinic.org/diseases‐conditions/high‐blood‐pressure/symptoms‐causes/syc‐20373410.	The reference is real; however, it is not directly related to the question “What is the prevalence of hypertension in the United States in 2019?”
Inaccurate^b^	Centers for Disease Control and Prevention (CDC). (2015). Prevalence of Amyotrophic Lateral Sclerosis—United States, 2015. MMWR. Morbidity and Mortality Weekly Report, 67(46), 1210–1214. https://www.cdc.gov/als/Default.html.	The author, title, and hyperlink are real; however, year of publication and pages are wrong (should be 2018: 67(46);1285–1289).
Incomplete^c^	American Cancer Society: Cancer Facts & Figures 2022. Atlanta, GA: American Cancer Society, 2022.	Without a specific hyperlink, the reference cannot be accessed.
Fabricated	Prevalence of Amyotrophic Lateral Sclerosis Among Non‐Hispanic Blacks in the United States, 1995–2015: A Population‐Based Study. Neurology. 2020;95(16):e2080–e2088. doi: 10.1212/WNL.0000000000010513.	Article does not exist. The journal, volume, and issue exist but pages do not exist. DOI is referred to an article irrelevant to the question.

^a^ Reference is real but not specific to the question and or fails to provide enough information for generating correct responses.

^b^ References without sufficient information to verify existence.

^c^ References identifiable via internet search but with incorrect author names, journal names, volume or page numbers, DOI, or hyperlinks.

## 4 Discussion

In this cross‐sectional study, we evaluated the performance of three LLMs in responding to questions on disease epidemiology. When comparing the results from LLMs with benchmark data, ChatGPT‐4 presented higher accuracy (76.2%) than Google Bard (50.0%) or ChatGPT‐3.5 (45.2%). When submitting the same questions to LLMs in queries on different dates, 71.4% of responses in ChatGPT‐4, 57.9% in Google Bard, and 46.7% in ChatGPT‐3.5 were consistent. We also found irrelevant, incomplete, inaccurate, and fabricated references in responses from ChatGPT‐4 and Google Bard, while ChatGPT‐3.5 did not generate any references.

To date, there have been no similar studies that assess the performance of LLMs in the field of pharmacoepidemiology. There has, however, been a growing body of research evaluating the performance of LLM‐based chatbots responding to questions related to various fields, such as pharmacology, patient education, and physician education [19, 20, 21, 22, 23, 24, 25]. In line with previous research [21, 23, 24, 25, 26, 27], our study has demonstrated that ChatGPT‐4 presented better overall performance over Google Bard and ChatGPT‐3.5. Unlike other studies, we observed higher accuracy for Google Bard in comparison to ChatGPT‐3.5. In a comparative analysis of the performance of three LLM‐chatbots in addressing 31 common myopia related queries, ChtaGPT‐4 showed highest accuracy (80.6%), followed by 61.3% in ChatGPT‐3.5 and 54.8% in Google Bard [23]. Coskun et al. found that GPT‐4 achieved an accuracy of 100% when answering methotrexate‐related questions for treating rheumatoid arthritis (e.g., mechanism of action, potential side effects) while ChatGPT‐3.5 scored 86.96% and Bard scored 60.87% [26]. The superior performance of ChatGPT‐4 may be attributed to its significantly larger number of parameters used in training than those in other LLMs. However, there was some evidence that ChatGPT‐4 was not always superior to the other LLMs. A better performance for Bard in answering questions related to drug–drug interactions than ChatGPT‐4 or ChatGPT‐3.5 was reported [22]. An important difference between Bard and ChatGPT lies in Bard's ability to access and incorporate real‐time information from the internet, while ChatGPT is not directly connected to the internet and relies on pre‐trained data to generate responses. The performance of these LLMs may also depend on the domain of training data, which may not fully cover disease epidemiology. To explore this further, we performed an exploratory analysis by providing ChatGPT‐4 with reference materials containing benchmark data, and then submitted questions on disease epidemiology. We found that the accuracy of responses increased to 89.5% (Table S10). This indicates that it is possible to address the information scarcity issue within LLMs by leveraging human expertise and knowledge.

Thus, the role of expert pharmacoepidemiologists is somewhat protected. In the first instance, our study has demonstrated that LLMs do not provide data that are of sufficient quality to use in drug safety evaluations. Similar to other studies, we found that LLMs generated inconsistent responses to the same question submitted in different queries [21, 28]. The lack of consistency in the results requires additional effort to verify the responses of LLMs. It also prevents other epidemiologists or regulatory agencies from replicating findings obtained from LLMs' outputs. A further limitation of LLMs is the fabrication of a plausible response containing factual errors or completely made‐up contents [5]. In our study, we observed inaccurate and fabricated references in Bard, which was also described in the literature [29, 30]. Aiumtrakul et al. reported that when tasked with providing references pertaining to nephrology, Bard provided 23% inaccurate, 63% fabricated, 11% incomplete references, and only 3% accurate references [31]. Although we did not observe inaccurate or fabricated references for ChatGPT‐4, possibly due to small sample size, high rates of fabricated and inaccurate references have been observed in other studies [11, 31, 32].

These findings emphasize the need to proceed cautiously with the use of LLMs in securing data for pharmacoepidemiologic purposes. Given our observed results, our recommendation is that the pharmacoepidemiologists working in a drug safety setting in the pharmaceutical industry must continue to perform a traditional literature search or design a bespoke study to obtain reference epidemiological data. Our effort to “train” the models demonstrates the need for pharmacoepidemiologic expertise because the pharmacoepidemiologists must have already retrieved good quality data to “feed” to LLMs so that LLMs can read and provide a summarized answer.

### 4.1 Strengths and Limitations

Our study represents one of the first studies to assess the utility of LLMs in pharmacoepidemiology research, in terms of accuracy, consistency, and authenticity. Our study offered valuable and practical insights into the feasibility and accuracy of LLMs in retrieving frequency of disease epidemiological data. Results from our study could help researchers understand the potential benefits and risks of LLMs in the drug safety setting. However, our study has limitations due to its focus on disease occurrence in the United States. In addition, only a few diseases were included in this study and the analysis was performed solely in English. Future research that evaluates the performance of LLMs' responses on a wider range of diseases, in non‐US countries, and in non‐English settings may help gain a broader understanding of the application of LLMs in epidemiologic research globally. Repeating the exercise in several months would also be of interest as the intelligence of chatbots increases.

## 5 Conclusion

In this cross‐sectional study, ChatGPT‐4 presented relatively better performance in retrieving information on disease epidemiology compared to Google Bard and ChatGPT‐3.5. However, all three LLMs presented inaccuracies in their responses that cannot be ignored, including irrelevant, incomplete, inaccurate, or fabricated references.

Our findings highlight the importance of retaining pharmacoepidemiology expertise in the drug safety setting to ensure the use of accurate and high‐quality data in observed versus expected calculations that support signal evaluations. While the use of LLMs seems attractive in expediting retrieval of epidemiology data, the verification process required will introduce workload and delays for epidemiologists seeking rapid information on disease epidemiology. This implies that LLMs may not enhance the quality or efficiency of pharmacoepidemiologic research in supporting drug safety evaluations.

Furthermore, the training data for LLMs lacks transparency, and there is a shortage of domain specific LLMs trained to enhance accuracy and consistency in information pertaining to disease epidemiology. “Feeding” the models with good quality reference materials still requires expertise in the first instance, for example in the ability to critically appraise the burgeoning literature and separate high‐quality studies from others.

As LLMs continue to progress and refine, continual evaluation of their strengths, risks, and application in pharmacoepidemiologic research over time is warranted.

### 5.1 Plain Language Summary

LLMs are generative AI applications that can produce human‐like responses to diverse queries. But can they provide accurate information on background epidemiology of diseases, which are required in pharmacoepidemiologic research? We designed 21 questions on the prevalence and incidence of diseases and submitted them to three LLMs, including ChatGPT‐3.5, ChatGPT‐4, and Google Bard. We also obtained benchmark data from traditional manual literature review targeting “gold‐standard” references (e.g., government statistics, peer‐reviewed articles). The accuracy of LLMs was evaluated by comparing LLMs' responses to the benchmark data. Consistency was determined by comparing the responses to the same query submitted on different dates. The relevance and authenticity of references was evaluated. We found that ChatGPT‐4 presented higher accuracy and consistency in retrieving information on disease epidemiology compared to Bard and ChatGPT‐3.5. However, all three LLMs presented inaccurate responses, including irrelevant, incomplete, or fabricated references. These findings suggested that the accuracy and reliability of LLMs' responses are not of acceptable standards for those who work in pharmaceutical industry, in academia, or in the regulatory setting, for example in establishing background epidemiology of diseases for use in drug safety signal evaluations or comparative safety research.

## Author Contributions

Study design: K.Z. and S.J.S. Data collection and curation: K.Z. and S.J.S. Data analysis: K.Z. and S.J.S. Results interpretation and discussion: All authors. Writing – original draft preparation: K.Z. Writing – review and editing: All authors. Supervision: S.J.S.

## Disclosure

Findings from this study were presented in the International Society for Pharmacoepidemiology (ISPE) 2024 Mid‐Year Meeting, April 14–16, 2024, Orlando, Florida, USA.

## Ethics Statement

The authors have nothing to report.

## Conflicts of Interest

K.Z. receives a postdoctoral fellowship from Sanofi. A.K., M.E., W.A.B., J.J., C.J.R., and S.J.S. are Sanofi employees and may hold shares and/or stock options in the company. J.Z. was a Sanofi employee at the time of conducting this study.

## Supporting information


Data S1.
